# Atomic-scale analysis of cation ordering in reduced calcium titanate

**DOI:** 10.1038/s41598-017-15120-2

**Published:** 2017-11-03

**Authors:** Luying Li, Xiaokang Hu, Fan Jiang, Wenkui Jing, Cong Guo, Shuangfeng Jia, Yihua Gao, Jianbo Wang

**Affiliations:** 1 Center for Nanoscale Characterization and Devices, Wuhan National Laboratory for Optoelectronics, and School of Physics, Huazhong University of Science and Technology, Wuhan, 430074 China; 2 School of Physics and Technology, Center for Electron Microscopy, MOE Key Laboratory of Artificial Micro- and Nano-Structures and the Institute for Advanced Studies, Wuhan University, Wuhan, 430072 China

## Abstract

The phenomenon of cation ordering is closely related to certain physical properties of complex oxides, which necessitates the search of underlying structure-property relationship at atomic resolution. Here we study the superlattices within reduced calcium titanate single crystal micro-pillars, which are unexpected from the originally proposed atomic model. Bright and dark contrasts at alternating Ti double layers perpendicular to ***b*** axis are clearly observed, but show no signs in corresponding image simulations based on the proposed atomic model. The multi-dimensional chemical analyses at atomic resolution reveal periodic lower Ti concentrations at alternating Ti double layers perpendicular to ***b*** axis. The following *in-situ* heating experiment shows no phase transition at the reported *T*_c_ and temperature independence of the superlattices. The dimerization of the Ti-Ti bonds at neighboring double rutile-type chains within Ti puckered sheets are directly observed, which is found to be not disturbed by the cation ordering at alternating Ti double layers. The characterization of cation ordering of complex oxides from chemical and structural point of view at atomic resolution, and its reaction to temperature variations are important for further understanding their basic physical properties and exploiting potential applications.

## Introduction

The reduced alkali metal and alkaline earth metal titanium oxides show various electronic and magnetic properties, among which Li_1+*x*_Ti_2-*x*_O_4_ is most notable for its relatively high *T*_c_ (~13 K)^1^. In 2002, the stoichiometric spinel MgTi_2_O_4_ was reported to go through a metal to insulator phase transition at 260 K^2^, and an unusual helical Ti-Ti dimerization was identified in MgTi_2_O_4_ later on^3^. As the ternary reduced titanate formed by the most close alkali metal to Mg, CaTi_2_O_4_ was first synthesized and structurally characterized in 1956^4^, and single crystals of CaTi_2_O_4_ was successfully grown in 1998^5^. It is predicted that CaTi_2_O_4_ might experience a structural transition at 650 K (*T*_c_) based on the similar downward trend of magnetic susceptibility below *T*_c_^6^ as compared to MgTi_2_O_4_^7^.

The phenomenon of cation ordering has been investigated in a wide range of complex oxides including exploring related physical properties (dielectric, ferroelectric, magnetic, and electronic response, *etc*.)^8^ as well as finding the underlying structure-property relationships based on characterization of the superstructures. For example, the ordering of lithium ions and vacancies is found to be responsible for the monoclinic distortion and the stability of the layered Li_*x*_CoO_2_ structure upon lithium deintercalation^9^; the compressibilities of pseudobrookite-type MgTi_2_O_5_ are reported to be highly dependent on the degrees of Mg-Ti ordering under high pressure conditions^10^.

Traditionally, the analysis of the superstructure including cation ordering mainly relies on reciprocal characterization techniques like X-ray, neutron or electron diffraction^11,12^. Direct observation and quantitative characterization of superstructures at atomic resolution become possible thanks to the development of aberration corrector facilitated transmission electron microscopes (TEM)^13,14^. In this work, direct observation of superstructure in reduced calcium titanate is realized, which is unexpected for single-phase CaTi_2_O_4_. Based on simultaneous spectroscopical and spatial characterizations of the superstructure at sub-angstrom resolution, the observed superstructure is attributed to cation ordering along ***b*** axis. The study of temperature dependence of cation ordering and the atomic structure of CaTi_2_O_4_ as a whole across the expected *T*_c_, and the influence of cation ordering on Ti-Ti dimmerization would be helpful for further understanding related physical properties in CaTi_2_O_4_ and other complex oxides.

## Results and Discussion

**Figure 1:**
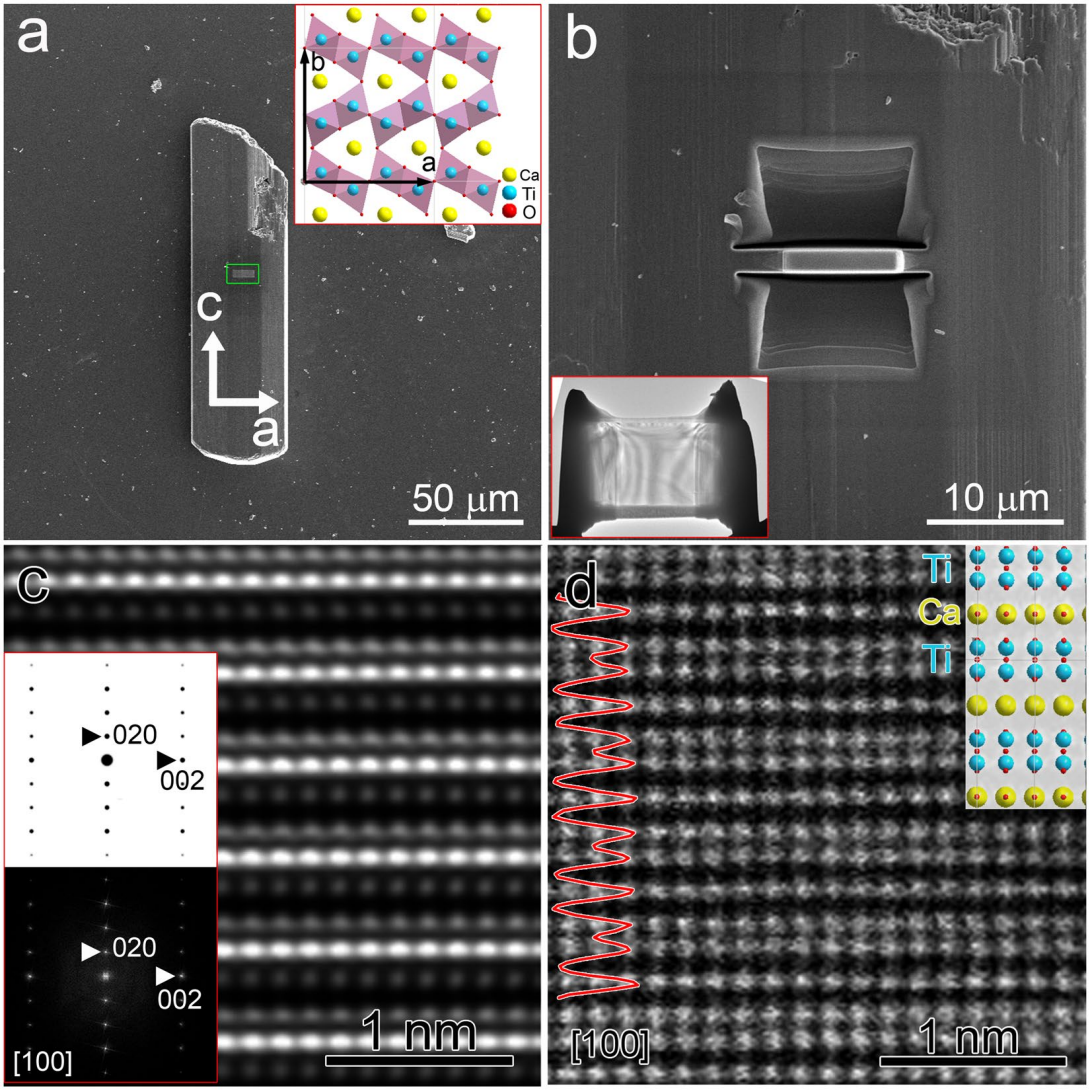
(**a**) SEM image of specific CaTi_2_O_4_ micro-pillar showing smooth surfaces, the region in a green box indicates Pt deposition as protective layer during FIB sample preparation. The up inset is atomic model of CaTi_2_O_4_ projected along [001]. (**b**) During sample preparation, trenches are formed on both sides of the thin piece, and the lower inset shows the final thinned sample with electron transparency. (**c**) HRTEM image of CaTi_2_O_4_ projected along [100], and the inset includes simulated (upper) and fast Fourier transform of the experimental HRTEM image (lower). (**d**) HAADF-STEM image of the same sample projected along [100]. The inset is atomic model projected in the same direction, and the red curve is intensity line profile of the HAADF image.

CaTi_2_O_4_ (space group: *Bbmm*) is composed of double-rutile chains of edge-sharing TiO_6_ octahedra, which are joined together by additional edge-sharing forming puckered TiO_6_ octahedra sheets perpendicular to the ***b*** axis. The neighboring sheets have mirror relations connected through corner-sharing. As a result, one-dimensional tunnels form extending along the ***c*** axis, where the calcium atoms are located, as shown in the upper inset of Fig. 1a. The lattice parameters of the orthorhombic unit cell are determined to be: ***a*** = 9.718 Å, ***b*** = 9.960 Å, ***c*** = 3.140 Å from single crystal X-ray diffraction data^5^.

Figure 1a is scanning electron microscopy (SEM) image of specific CaTi_2_O_4_ micro-pillar showing smooth surfaces, the longer edge of the pillar is along ***c*** axis. Pt film was deposited in the region labeled by a green box as protection layer during FIB thinning. With irradiation of a Ga probe, trenches were formed on both sides before the central thin piece was lifted out, as shown in Fig. 1b, and the final thin piece of electron transparency is presented in the lower inset of Fig. 1b. Two pieces perpendicular to ***a*** and ***c*** axes were prepared, respectively, using the same method.

The piece with surfaces perpendicular to ***a*** axis was characterized first by HRTEM and STEM-HAADF techniques. Figure 1c is HRTEM image of the piece projected along [100] direction, and the corresponding fast Fourier transform (FFT) (the lower inset with black background) shows typical diffraction pattern of CaTi_2_O_4_ along [100], which is the same as the calculated diffraction pattern (upper inset with white background) based on the unit cell atomic positions determined by X-ray powder diffraction^5^. The aberration-corrected HAADF image of the same piece projected along [100] is presented in Fig. 1d, the atomic model projected in the same direction is overlapped for comparison. It is obvious that the double atomic layers correspond to Ti layers, while the single atomic layer in between is composed of Ca atoms. The red curve on the left is intensity line profile of the HAADF image showing close intensities of Ti and Ca atomic columns (Z_ca_ = 20, Z_Ti_ = 22). Moreover, the HRTEM image and HAADF image at the same magnification resemble each other in their respective imaging conditions.

**Figure 2:**
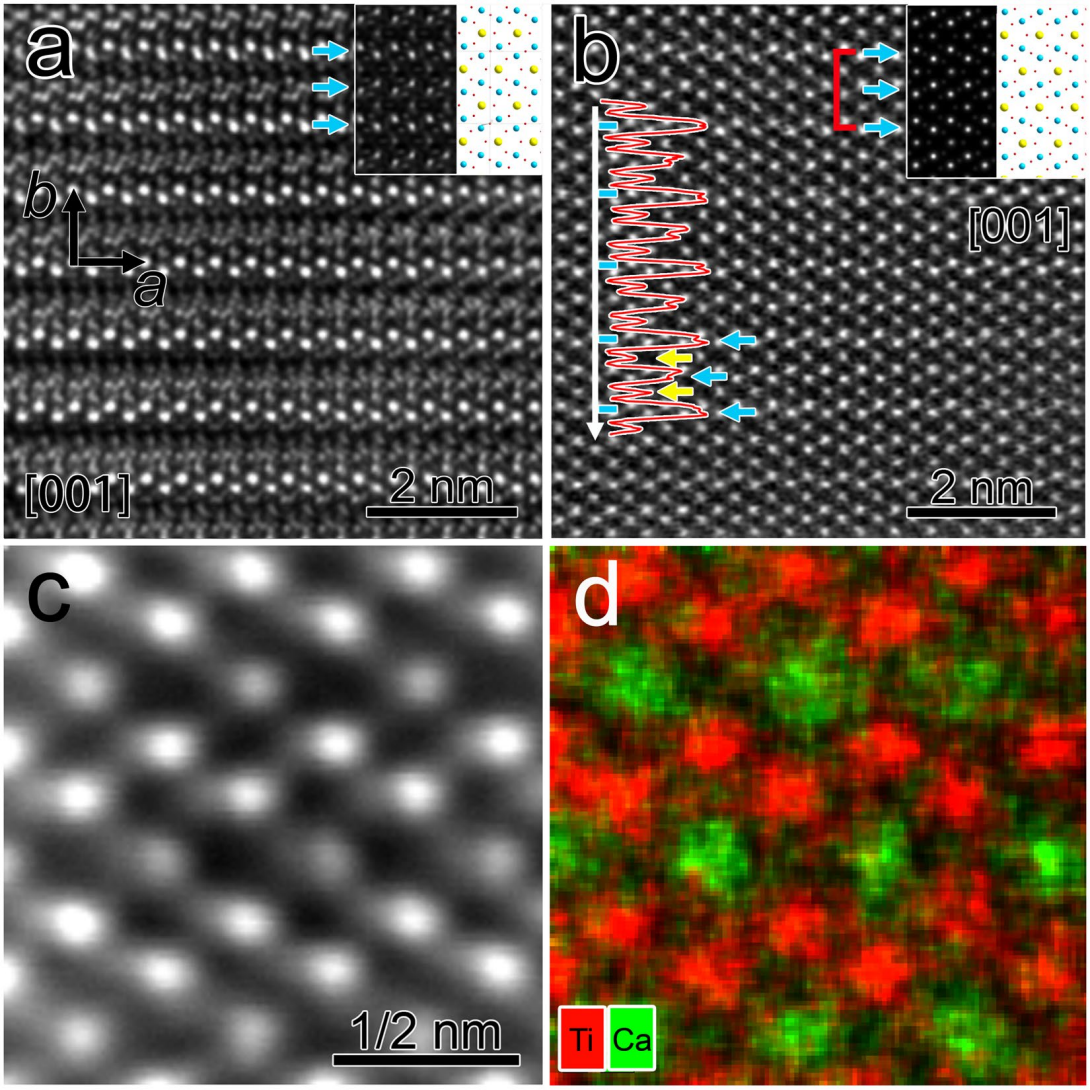
(**a**) HRTEM image of CaTi_2_O_4_ projected along [001]. The inset is atomic model of CaTi_2_O_4_ projected along the same direction (right) and simulated HRTEM image (left) based on the model. (**b**) HAADF-STEM image of CaTi_2_O_4_ projected along [001]. The inset is atomic model of CaTi_2_O_4_ projected along the same direction (right) and simulated HAADF image (left) based on the model. The blue and yellow arrows indicate the locations of puckered Ti layers and Ca layers, respectively. The red curve is intensity line profile of the HAADF image along the white arrow. (**c**) Atomic resolution HAADF image along [001] and (**d**) corresponding elemental map, the red and green spots represent Ti and Ca atomic columns, respectively.

The other piece perpendicular to ***c*** axis was characterized later by similar techniques. The HRTEM image of the piece projected along [001] direction is shown in Fig. 2a. With period of *a* for lattices along ***a*** axis as expected, the (010) planes perpendicular to ***b*** axis show alternating bright and dark contrasts leading to a period of 2b. The top inset is the atomic model of CaTi_2_O_4_ projected along [001] direction (right), and the corresponding simulated HRTEM image (left) is performed using JEMS software (defocus: −55 nm, thickness: 11.6 nm) and overlapped on the experimental image. It is apparent that the (010) planes in the simulated HRTRM image are of similar contrasts, as indicated by the blue arrows, without doubling of the period along ***b*** axis.

The aberration-corrected HAADF image projected along [001] is obtained from the same sample, as presented in Fig. 2b, which clearly shows the Ca and Ti atomic columns at atomic resolution. The top inset is arranged in the same way as that in Fig. 2a, and the simulation of HAADF image (left) is carried out employing MACTEMPAS software. The simulated image shows slightly higher intensities of the Ti columns than the Ca columns, and uniform contrasts for continuous (010) double Ti planes, as indicated by the blue arrows. Superstructure comprised of faint lines at the (010) planes with period of 2*b* in the experimental HAADF image is labeled by short blue lines attached to the long white arrow on the left. The corresponding intensity line profile across five periods of the superstructure along ***b*** axis is presented as a red curve, and overlapped to the experimental HAADF image. Since the two Ti layers composed of the double-rutile chains (labeled by blue arrows) are very close to each other, their projected intensities sum up and give an overall intensity of about twice that of the neighboring Ca layers (labeled by yellow arrows), and the double Ti layers show alternating high-low intensity peaks, with the faint lines corresponding to those higher peaks.

On the other hand, the intensity line profiles at Ti double layers along ***a*** axis show no obvious periodic intensity variations (Supplementary Figure S1a,b) and the three dimensional 3 × 3 supercell model (Supplementary Figure S2b) indicates non-uniform Ti atom densities along ***a*** axis. It is hard to determine whether the variations in the corresponding intensity line profiles along ***a*** axis are due to different Ti atom densities or possible existence of Ti vacancies. Thus, the superstructure is observed in both HRTEM and HAADF-STEM modes along ***b*** axis, which might be attributed to additional structural features introduced to the original atomic structural model.

**Figure 3:**
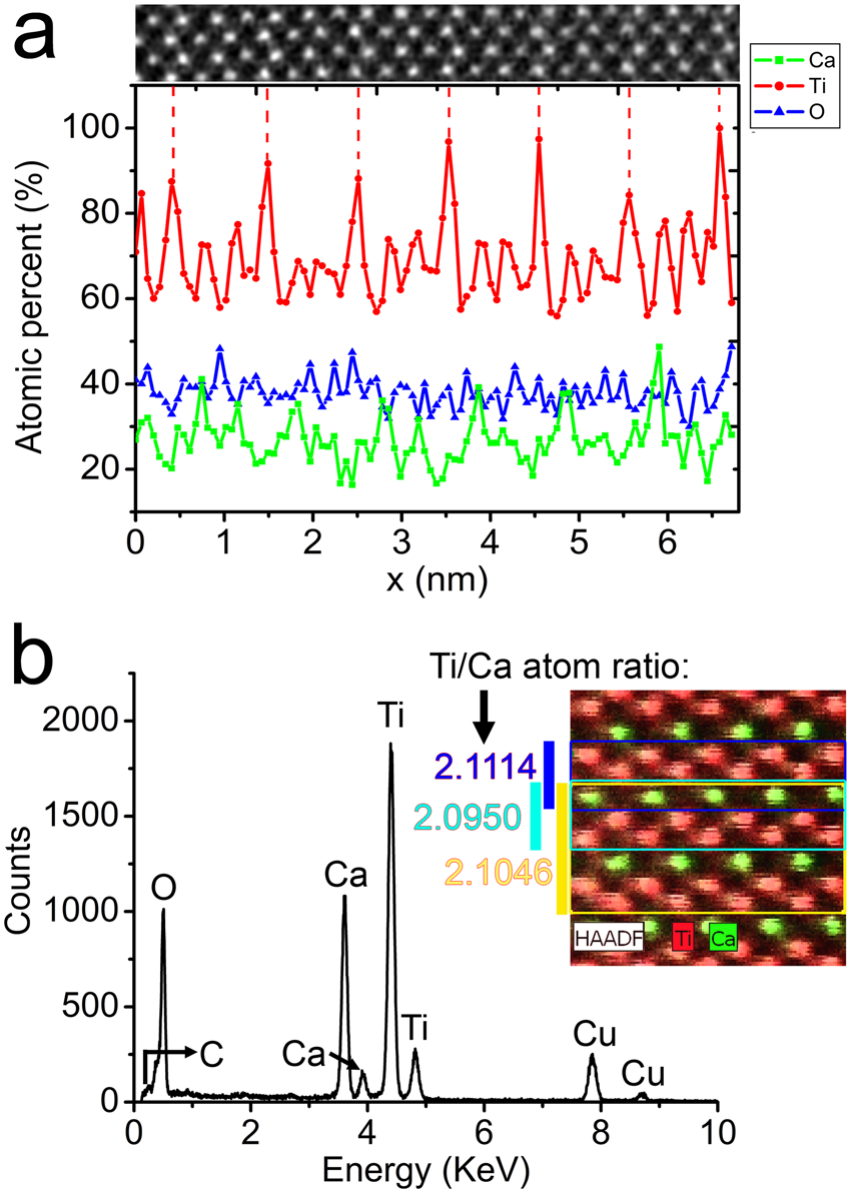
(**a**) Profiles of atomic percentages of Ca, Ti, O in respective colors. The HAADF image of the scanned region for chemical analyses is added on top for comparison. (**b**) EDS spectrum of CaTi_2_O_4_. The top inset is elemental map of the region used for local atom ratio quantification.

To figure out the origin of the superstructure in CaTi_2_O_4_ micro-pillars, the chemical information is obtained at atomic resolution. Figure 2c is HAADF image with [001] projection of a region used for chemical analysis, and Fig. 2d is corresponding elemental maps, where the red and green spots represent Ti and Ca atomic columns, respectively. The puckered double Ti layers and single Ca layers in between are clearly shown as expected. The atomic percentages of constituent elements (Ca, Ti, and O) across selected region including six periods of the superstructure are shown in respective colors in Fig. 3a, and the HAADF image of the scanned region for elemental analysis is added on top for comparison. The profile for Ti shows periodic higher peaks right at the position of the faint lines. Although EDS is not excel in quantification of elemental concentration without calibration with reference samples, the periodic variations of Ti concentration across the superstructure indicate higher Ti concentrations at the Ti layers showing faint lines in the HAADF image, and lower Ti concentrations at the Ti layers in between.

Figure 3b is EDS spectrum of CaTi_2_O_4_ showing the existence of Ca, Ti, O without traces of any impurity dopants. The C and Cu signals should be resulted from surface contaminants and the copper grid as supporter. The top inset in Fig. 3b is elemental map of specific region where the Ti/Ca atom ratios are measured for certain atomic layers. The blue box includes one puckered Ti double-layers (at the faint line) and the neighboring Ca layer, the corresponding atom ratio is measured to be 2.1114. The cyan box contains the same Ca layer and the puckered Ti double-layers (between faint lines) below, which gives an atom ratio of 2.0950. The yellow box region corresponds to one period of the superstructure with an atom ratio of 2.1046, which is very close to the average value of the above two. Although the absolute values of the measured atom concentrations might not be entirely reliable, the chemical analyses carried out at different areas give similar trend of elemental distributions. Thus, it can be concluded that the superstructure along ***b*** axis is mainly attributed to periodic Ti deficiencies within alternating puckered Ti double-layers.

With no signatures of other impurity dopants of lighter elements from EDS measurements, the possibility of substitution of Ti positions for lighter elements is precluded. Since the Ti/Ca atom ratio between the faint lines is slightly lower than that right at the faint lines, it is quite possible that Ti vacancies appear in alternating Ti double layers, which contributes to smaller average atomic number for those Ti layers with lower intensity in the HAADF image. Close inspection of the projected Ti-Ti bond lengths reveals no difference for the alternating puckered Ti double-layers. Thus, there should not be large amount of Ti vacancies, with possible collapse of the double rutile chains, appearing in the puckered Ti double-layers. Since the scattered intensity for HAADF imaging is proportional to projected sample thickness, the Ti vacancy rate is evaluated from intensity line profile across single atomic columns along ***b*** axis, which gives a value of 9.86% for Ti double layers including vacancies.

**Figure 4:**
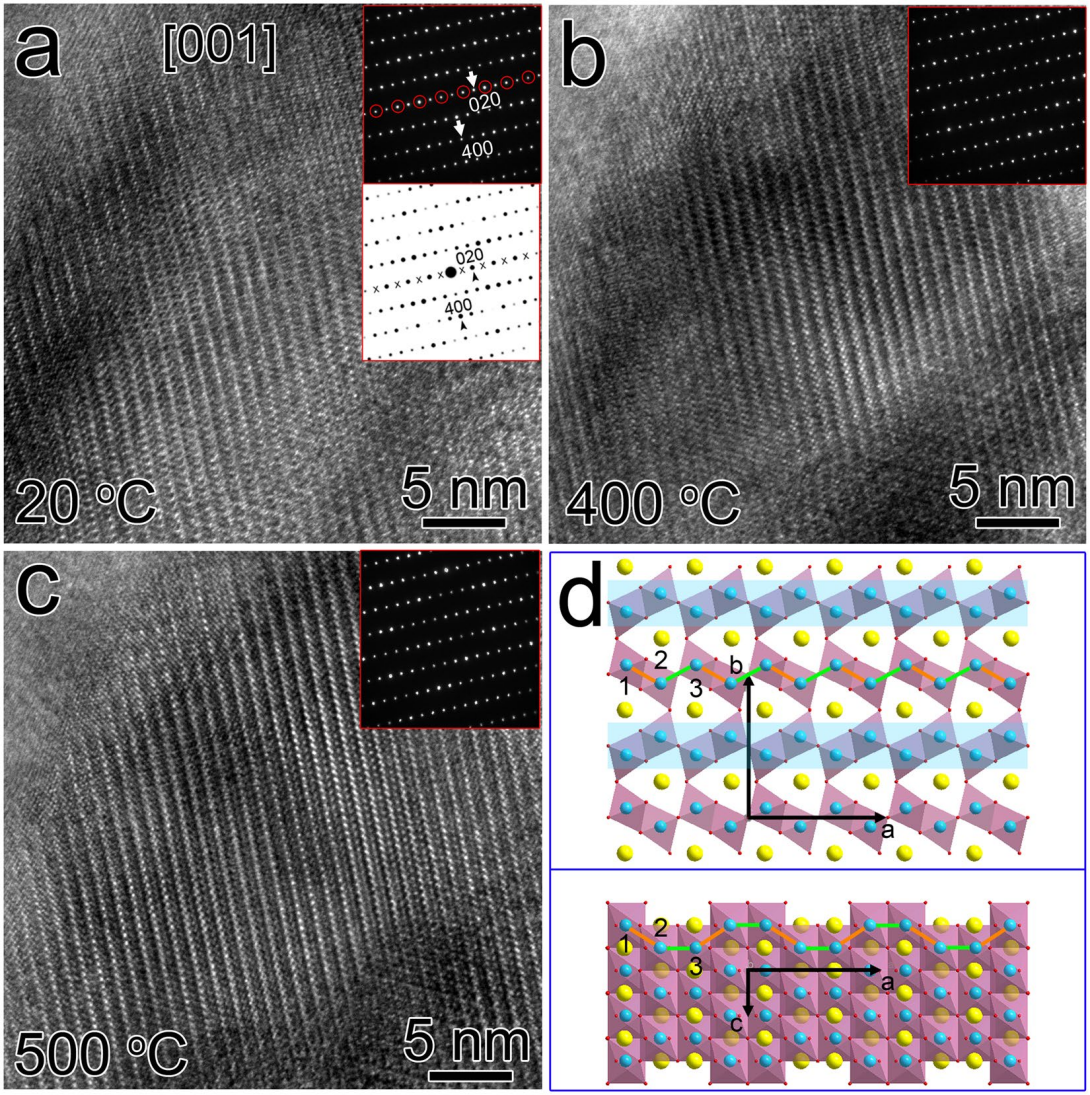
Study of the temperature dependence of the superstructure. The HRTEM images show specific region in CaTi_2_O_4_ projected along [001] at (**a**) 20 °C, (**b**) 400 °C and (**c**) 500 °C, respectively. The top insets with black background are corresponding experimental SAED patterns, and the lower inset with white background is simulated SAED pattern based on the original atomic model. (**d**) The original model projected along [001] and [010]. The orange and green lines indicate different bond lengths in the puckered double Ti layer. The Ti double layers covered by blue bands in the top model indicate the existence of Ti vacancies in alternating Ti double layers.

The dependence of the superstructure on temperature is also studied. Considering the suggested structural transition of CaTi_2_O_4_ at ~650 K (377 °C, *T*_c_)^6^, the same sample with surface normal along [001] is heated from 20 °C (room temperature) to 500 °C. Figure 4a–c shows HRTEM images of specific area at 20 °C, 400 °C and 500 °C, respectively. The upper insets with black background are corresponding SAED patterns, the (020) and (400) diffraction spots are labeled, and the (0*k*0) (*k* = ±1, ±3, ±5…) spots are circled out for clarity. The SAED pattern with white background is simulation along the same direction based on the original atomic model, where the (0*k*0) reflections are kinematically forbidden. The appearance of the (0*k*0) reflections in the experimental SAED patterns could be attributed to cation ordering in alternating Ti double-layers in the form of Ti vacancies.

As the temperature increases, the crystal lattices do not have obvious differences among different temperatures, and all of the corresponding SAED patterns clearly show (0*k*0) reflections, indicating the existence of superstructures. Thus, the Ti vacancy induced cation ordering in alternating Ti double layers seems to be very stable at variable temperature conditions, which is different from the case of superstructures in potassium tungsten bronze nanosheets, where the W vacancies become more uniformly distributed at higher temperature^15^. Moreover, the suggested phase transition at ~377 °C^6^ does not happen in the current case. It is possible that the existence of cation ordering hinders the transition around *T*_c_, or the observed downward trend of magnetic susceptibility below *T*_c_ is resulted from other factors rather than phase transition as the case of MgTi_2_O_4_.

As mentioned above, a helical dimerization pattern was reported in MgTi_2_O_4_, which would result in orbital ordering and the opening of a gap between the fully occupied and unoccupied states^3^. The existence of Ti-Ti dimmers in CaTi_2_O_4_ was also proposed from indirect experimental proofs^6,16^. In the standard CaTi_2_O_4_ atomic model, the Ti-Ti distances within double rutile-type chains are 3.135 Å and 3.140 Å, while the Ti-Ti distance between Ti atoms in neighboring double rutile-type chains within a puckered sheet is 2.784 Å^6^. Figure 4d shows the single crystal model of CaTi_2_O_4_ projected along ***c*** (top) and ***b*** (bottom) axes. The orange and green lines indicate Ti-Ti bonds within a double rutile-type chain, and between two neighboring chains, respectively, and the two types of bonds are of similar length as observed from ***c*** axis projection. When projected along ***b*** axis, the Ti atoms labeled as 1, 2, 3 in the top model are found to be at different heights along c axis. Thus, the length of orange bonds in the top model is projected length of the actually bonds.

The green and orange arrows in Supplementary Figure S1a label lines of atoms used for intensity profiles in Supplementary Figure S1c, and the types of atoms corresponding to the intensity peaks are added on top of Supplementary Figure S1c for clarity. It is found that the green Ti bond lengths are slightly larger than the orange bonds, which are measured to be 2.717 Å (orange bonds) and 2.765 Å (green bonds), respectively. After taking the projected bond angles into consideration, the actual bond lengths are 3.139 Å (orange) and 2.765 Å (green), which are very close to the reported values. Moreover, the blue rectangles in Supplementary Figure S1c label Ti double layers including possible Ti vacancies, and the green double arrows are of the same length, indicating similar Ti-Ti bond length at alternating regular Ti double layers and layers including Ti vacancies in between. Thus, the dimerization in CaTi_2_O_4_ micro-pillars is clearly verified, which is not obviously affected by the existence of cation ordering in alternating Ti double layers perpendicular to ***b*** axis.

## Conclusion

CaTi_2_O_4_ micro-pillar single crystals are successfully synthesized. While the CaTi_2_O_4_ crystal projected along ***a*** axis has the same structure as the proposed atomic model, the same crystal projected along ***c*** axis shows superstructures of bright and dark contrasts at alternating Ti double layers perpendicular to ***b*** axis, which is unexpected from both HRTEM and HAADF image simulations based on the original atomic model. Further EDS chemical analyses at atomic resolution preclude the existence of lighter elements as dopants, and reveal lower Ti concentrations at Ti double layers between the faint lines. The following *in-situ* heating experiment shows no obvious phase transition at the expected *T*_c_, and the cation ordering related superstructure is found to be temperature independent. Moreover, the dimerization of Ti-Ti bonds is clearly observed, which is not affected by cation ordering as well. Study of cation ordering in CaTi_2_O_4_ from chemical and structural point of view at atomic resolution provides valuable information for tuning of cation ordering in CaTi_2_O_4_ and related complex oxides, and for further understanding of their related physical properties.

## Methods

Single crystals of CaTi_2_O_4_ under study were prepared by the molten salt flux method. Equi-molar quantities of Ti metal powder (>99%) and CaTiO_3_ powder (>99%) were ground together with CaCl_2_ powder on the surface to prevent oxidation of the reactants. The temperature was quickly raised to 1000 °C at 130 °C/h and kept at 1000 °C for 6 h, and then cooled to 700 °C at 0.5 °C/min. The resultants were washed by deionized water to remove CaCl_2_, and the suspension was sonicated to separate the leftover. The comparatively larger crystals at bottom were CaTi_2_O_4_^16^.

The obtained CaTi_2_O_4_ single crystals are mostly micro-pillars with length in the range of 50~500 μm. Small pieces along certain orientations are cut out from specific CaTi_2_O_4_ micro-pillar and thinned by FEI Quanta 3D FEG focused ion beam (FIB) for TEM observation. The high-resolution transmission electron microscopy (HRTEM) images, and aberration-corrected high-angle annular-dark-field (HAADF) images were obtained using FEI Titan G^2^ 60–300 with a probe Cs corrector operating at 300 kV. With a C2 aperture of 70 μm, the corresponding convergence semi-angle was ~28.8°. The atomic spectroscopic analysis was carried out using FEI Titan ChemiSTEM operating at 200 kV with the detector super EDS energy resolution ≤130 ev/100 kcps. The *in situ* heating experiment was completed using DENSsolution-DH30-4M-JU double-tilt heating/biasing holder.

## Electronic supplementary material

Supplementary Information
