# Supplementary material for: Atomic-scale analysis of cation ordering in reduced calcium titanate
Source: Sci Rep. 2017 Nov 3;7:14977. doi: 10.1038/s41598-017-15120-2 (PMC5670233; doi:10.1038/s41598-017-15120-2)
Supplement: Supplementary file 1 — Supplementary Information [file 41598_2017_15120_MOESM1_ESM.doc]

Supplementary Information

**Atomic-scale analysis of cation ordering in reduced calcium titanate**

Luying Li^1,*^, Xiaokang Hu^1^, Fan Jiang^1^, Wenkui Jing^1^, Cong Guo^2^, Shuangfeng Jia^2^, Yihua Gao^1^, and Jianbo Wang^2^

^1^Center for Nanoscale Characterization and Devices, Wuhan National Laboratory for Optoelectronics, and School of Physics, Huazhong University of Science and Technology, Wuhan 430074, China

^2^School of Physics and Technology, Center for Electron Microscopy and MOE Key Laboratory of Artificial Micro- and Nano-Structures, Wuhan University, Wuhan 430072, China

*[luying.li@hust.edu.cn](mailto:luying.li@hust.edu.cn)


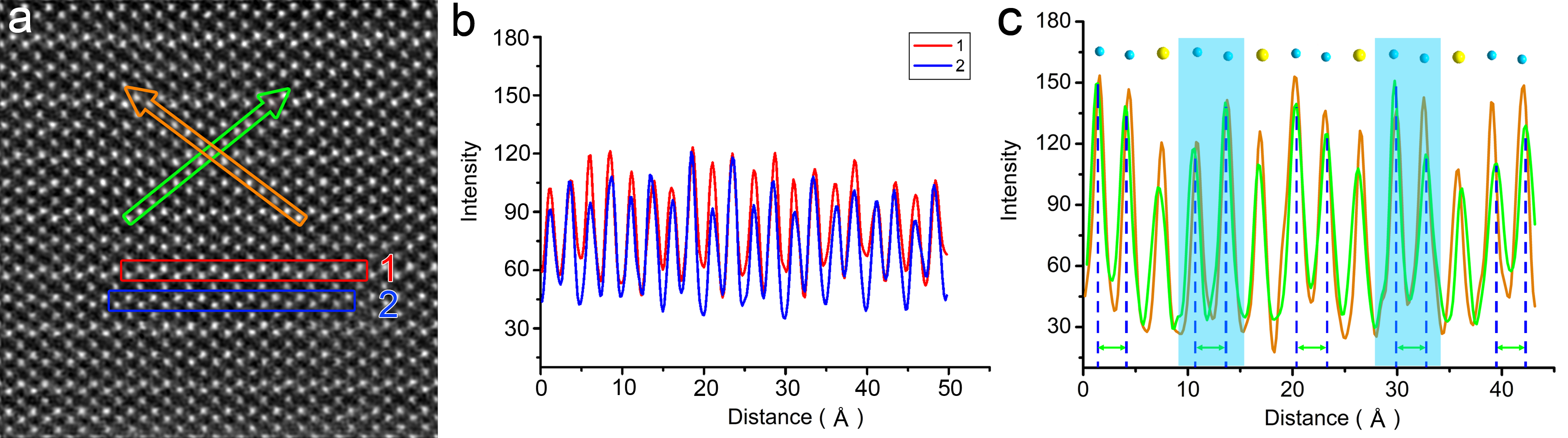


**Figure** S1. (a) High resolution HAADF STEM image used for intensity line profiles (the same as Figure 2b). Intensity line profiles of Ti double layers along ***a*** axis are labeled by red and blue rectangles in (a), and the results are shown in (b) in respective colors. Intensity line profiles across the atomic arrays labeled by green and orange arrows are presented in (c) in respective colors. The atomic model of the atomic arrays are added on top for clarity, the blue rectangles indicate Ti double layers including Ti vacancies, and the green double-head arrows show Ti-Ti bonds of the same length.


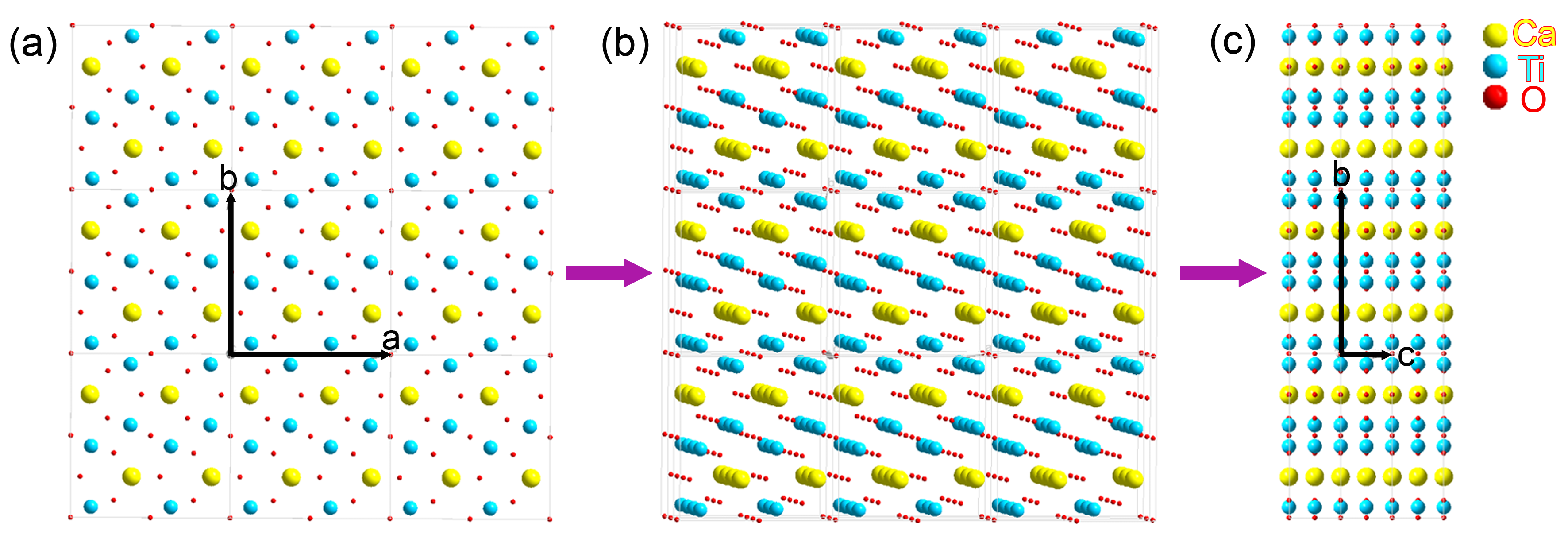


**Figure** S2. (a) 3×3 supercell model of CaTi_2_O_4_ projected in [001] direction. (b) The supercell model rotated slightly around ***b*** axis to show the three dimensional atomic arrangements. (c) The supercell model projected in [100] direction.
